# Supplementary material for: Learning curve and surgical outcome of robotic assisted colorectal surgery with ERAS program
Source: Sci Rep. 2022 Nov 29;12:20566. doi: 10.1038/s41598-022-24665-w (PMC9709162; doi:10.1038/s41598-022-24665-w)
Supplement: Supplementary file 2 — Supplementary Information 2. [file 41598_2022_24665_MOESM2_ESM.zip › RAL Raw data-Ñ[▒K/111/OP N_ñw╝╨░O▒Kñσ.pdf]

W52 / W52

臺中榮民總醫院

手術室護理記錄

[redacted]

基本資料

- 室別： 開刀房-17室，手術科別： CRS，手術類別： 常規，日期： 2021/09/09
- 進入時間： 1455 ； 麻醉開始時間： 1510 ； Time Out時間： 1600 ； 劃刀時間： 1601 ； 手術結束時間： 2210 送出時間： 2215 ；
- 麻醉方式： GE

  診斷：Colon polyps

  手術名稱：Robot Assisted Low Anterior Resection

  手術局部用藥：NIL.

  術前預防性抗生素：
  - 抗生素名稱一： Veterin 1GM IVD由麻醉護理師給予
  - 時間： 1545
  - 上次簽章: 張秀敏

  ，追加預防性抗生素：
  - 抗生素名稱一： Veterin 1GM IVD由麻醉護理師給予
  - 時間： 1955
  - 上次簽章: 張秀敏
- 手術醫師： 蔣鋒帆
- 麻醉醫師： VS 齊光彪
-
  - 刷手護士A ： 許芷瑄，時間： 1455，刷手護士B ： 盧陵怡，時間： 1455，接替刷手一： 許雅芳，時間： 2000，接替刷手二： 林靖容，時間： 2200
  - 流動護士A ： 張秀敏，時間： 1455，接替流動一： 盧陵怡，時間： 1630

皮膚狀況

A-V Shunt： 無

術前： 完整

記錄者： 上次簽章: 張秀敏

術後： 完整

記錄者： 張秀敏

手術準備

手術臥式 單一手術臥式

-
  - 手術臥式： 截石臥式
  - 使用附件： 溫毯 38 ℃，頭圈，腳架，其它： 廠商擺位用物
  - 皮膚準備： 10%Aq Betadine，Hibiscrub，2% Chlorhexidine
  - 電燒： 使用電燒: 電極片位置： 其它： 迴路電極墊，記錄者： 上次簽章: 張秀敏

止血帶T： 無

植入物

- 植入物名稱/號碼/數量/部位：ROD/ileostomy

，記錄者： 張秀敏

標本送檢

- 細菌培養 無
- 細胞學 無
- 冰凍切片 無
- 病理標本

  組織名稱一：distal cut end

  ,

  組織名稱二：rectum

  ,

組織名稱三： IMA Root

- 截肢 無

標本交付 標本： 3 袋 , 標本： 0 盒 , 標本： 0 瓶 , 細胞學： 0 管 , Culture Tube： 0 管 , 被交付醫師： R 洪浚嚴 ,

- 記錄者： 上次簽章: 張秀敏
- 時間： 2000

傷口術後敷料

傷口敷料： 傷口敷料： 無

- 請於圖上標示部位：

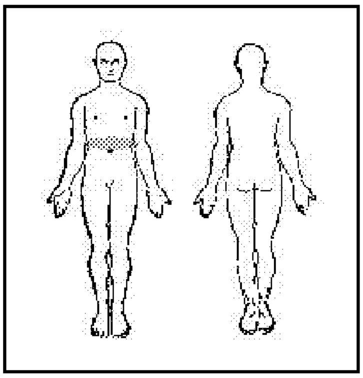

- 傷口敷料： 紗布

導管

導管	天數	導管資訊	插入日期	插入時間	更換日期	拔除日期	拔除時間	拔除原因	預計拔除/更換	預計拔除/更換日期
Foley(1)	1	號碼:16 Fr.; 部位:膀胱;	2021/09/09	1520						
Double Lumen CVC(1)	1	號碼:8 Fr.; 深度:15cm; 部位:jugular (右); 象限:RU;	2021/09/09	1515						
Epidural PCA(1)	1	部位:硬脊膜外;	2021/09/09	1450						
Sump NG Tube(1)	1	號碼:16 Fr.; 深度:60cm; 部位:胃;	2021/09/09	1607						
A-line (1)	1	號碼:20 G; 部位:左手;	2021/09/09	1510						

用物計數

- 手術中出血量： 100 c.c.
- 手術中尿量： 650 c.c.
-
  - 其它： 無

用物計數： 多切口同時縫合

- 2.縫針： 正確 , 3.刀片： 正確 , 4.紗布： 正確 , 5.腹部墊： 未使用 , 7.PEANUTS ： 未使用 , 8.紗條： 未使用 , 9.絲帶： 未使用 , 10.針頭： 未使用 , 11.Vessel loop： 未使用 , 12.Boots： 未使用 , 13.Jelco： 未使用 , 14.棉條： 未使用 ,
  - 第一次：
    - 刷手護士 ： 許雅芳
    - 流動護士： 上次簽章: 張秀敏
    - 時間： 2030

,

  - 第二次：
    - 刷手護士 ： 許雅芳
    - 流動護士： 上次簽章: 張秀敏
    - 時間： 2035

,

  - 第三次：
    - 刷手護士 ： 許雅芳

- 流動護士： 上次簽章: 張秀敏
- 時間： 2040

備註

備註： H2O200092109082176

- 記錄者： 上次簽章: 張秀敏
- 時間： 1600

填寫護理人員 張秀敏

正式摘要

W52 / W52

doctorRank:B
